# Implementation of a Biorisk Management System in Armenia for ISO 35001:2019 Certification

**DOI:** 10.1089/apb.2025.0007

**Published:** 2025-04-21

**Authors:** Hrant Danelyan, Pertsh Tumanyan

**Affiliations:** Reference Laboratory for Especially Dangerous Pathogens (RLEDP), Food Safety Inspection Body, Republic of Armenia, Yerevan, Armenia.

**Keywords:** Armenia, ISO 35001:2019, Biorisk Management System, risk assessment, veterinary laboratories, biosafety and biosecurity

## Abstract

**Introduction:**

Infectious disease outbreaks present significant public health and economic challenges that continue to persist worldwide. Accidental laboratory release or criminal misuse of infectious agents adds to these challenges, underscoring the need for responsible research and adequate biological risk (biorisk) mitigation. Countries such as Armenia that lack comprehensive legal frameworks for biosafety and biosecurity are proactively adopting standards such as International Organization for Standardization (ISO) 35001:2019 to enhance their safety culture and build a strong foundation through a structured Biorisk Management System. This study aimed to evaluate the biorisk management gaps at the Reference Laboratory of Especially Dangerous Pathogens (RLEDP) and implement the necessary steps to achieve ISO 35001:2019 certification.

**Methods:**

RLEDP worked with both the Management Systems and Business Consultants to perform a gap analysis of the current situation at RLEDP as it pertains to biorisk management, and with international subject matter experts through the Defense Threat Reduction Agency Biological Threat Reduction Program (BTRP) program to provide guidance and training to assist RLEDP.

**Results:**

The gap analysis highlighted areas that needed improvement, such as documentation, risk assessment methodologies, and staff training. The analyses and requirements led to the development and/or finalization of 11 facility-specific documents and the completion of 40 individual training courses for RLEDP staff. This work culminated in the development of a Biorisk Management Plan and ISO 35001:2019 certification.

**Conclusions:**

In 2022, RLEDP achieved ISO 35001:2019 certification and passed its first annual audit in 2023, demonstrating its commitment to biosafety and biosecurity. Continuous updates, interdepartmental collaboration, and legislative improvements remain essential for maintaining high standards.

## Introduction

Infectious disease outbreaks represent serious public health and economic challenges that should be diligently identified, as they can cause loss of life, disrupt international trade, and lead to restrictions on the movement of people and animals. Another critical factor is the use of infectious agents for criminal purposes. The rise of the use of pathogens in adversarial attacks with dual-use concerns reminds those working responsibly with these agents of the importance of the risks involved and the consideration for research reported.^1^ The responsibility for preventing such situations and containing potential damage lies with national governments and at the local level, but infectious diseases can be spread worldwide, so the role of international organizations in coordinating synchronized implementation of the work at the global level is also crucial.^2^ Laboratories play a vast role in the fight against infectious diseases, as they represent an important link in the chain of infectious disease control. In addition to diagnostic activities, laboratories are also an important part of education, research, and science and industry technologies.^3^ However, despite their importance, laboratories represent a source of biological hazards that can pose a potential danger to the general population and the environment, directly through laboratory staff. Therefore, laboratories must take appropriate measures to ensure that their biosafety and biosecurity activities are safe and in accordance with specified standards in order to minimize and neutralize existing risks.^4^ The Global Health Security Agenda and the International Health Regulations also play a significant role in improving biological (biorisk) management and implementation.^5^ These rules, guidelines, and requirements, coupled with national regulations, can support laboratories to improve biorisk management based on various biosafety levels (BSLs),^6–11^ but there are still disconnects in the definition of “internationally accepted standards,” including variations that can exist within countries.^12^ Additionally, many countries, including Armenia, whose legislation is still in progress, are also lacking in distinct legal frameworks for biosafety and biosecurity on a national level.^13^

Recognizing the importance of laboratories in the fight against infectious diseases and the need to ensure safe laboratory activities, laboratories are now beginning to evaluate their risks and implement a new or updated Biorisk Management System in accordance with the International Organization for Standardization (ISO) 35001:2019 standard requirements to manage biological threats.^14,15^

A Biorisk Management System is an important component of the general management system of many medical, science, and research organizations. It is a valuable tool that allows an organization to increase its biorisk capacities, utilizing a continual process of identification, assessment, control, and evaluation of the biosafety and biosecurity risks that exist or may occur in the organization. The Biorisk Management System is an ongoing development system that cycles through planning, implementing, reviewing, and improving specific activities through the Plan-Do-Check-Act (PDCA) principle,^16,17^ which can be continually improved.

Measures of biosafety and biosecurity are essential at all levels of a facility, which includes laboratory spaces, and should be considered at every step of the process. These measures should begin during the design and construction of the facility and continue when supplying the laboratory with modern equipment and safety protocols, when providing appropriate training measures to all staff, and during the implementation of working procedures. A combined understanding of the implementation of these measures is designed to reduce the potential impact of pathogens on the environment and on human and animal populations beyond the laboratory. However, these safety measures also include implementation of priority biosafety and biosecurity measures within the laboratories to protect staff from pathogen exposure and possibly causing laboratory-acquired infections (LAIs).^2,18,19^ Laboratory facilities must not only safely contain various pathogens within the laboratory spaces, but the development of LAIs in staff can lead to a chain of events outside of the laboratory when transmissible agents are involved, leading to human-to-human^20^ with the potential for human-to-animal transfer.^21^ Proper safety and maintenance of records can be imperative when confirming LAIs and implementing the proper safety protocols.^22^ Without these safeguards, and occasionally with the safeguards, there are still numerous instances of LAIs in laboratory staff who are unaware of exposure.^22,23^

Ensuring safe and secure activities in the laboratories is becoming difficult in parallel to increasing the BSL of the laboratory, and especially the use of increasing hazardous agents in BSL-2, BSL-3, and BSL-4 laboratories.^2^ The competence of laboratory personnel is an important prerequisite for preventing and reducing possible risks of infection and the spread of LAIs, as inexperienced laboratory staff can increase the possibility of biological accidents and acquiring an LAI even when the appropriate building and equipment are available and proper safety procedures are in place.^2^ Competent staff must also be acutely aware of the biohazards with which they are working and understand their consequences through extensive training and education.^18^

Therefore, the goal of this study was to define gaps in the Reference Laboratory for Especially Dangerous Pathogens (RLEDP) against ISO 35001:2019 and take steps to ensure compliance with this international standard. In Armenia, the RLEDP operates in the veterinary sphere, including veterinary laboratory diagnostics, training, and education, and is dynamically involved in active and passive epidemiological surveillance activities of infectious diseases, including those that are zoonotic. This surveillance includes conducting laboratory screening for unknown diseases, as well as diagnostic and monitoring investigations for diseases endemic to Armenia, which makes improving the Biorisk Management System of the RLEDP imperative for safety, accountability, and traceability.

## Materials and Methods

### Study Design

The process of improving the present Biorisk Management System in the RLEDP and for implementation of the ISO 35001:2019 standard started with the creation of a working group, which included the head of the laboratory, the chair of the committee of biosafety and biosecurity, the responsible official for biosafety and biosecurity, the heads of each of three departments, and consultants from Management Systems and Business Consultants (MSBC). Then, an MSBC consultant performed a gap analysis using the ISO 35001:2019 checklist covering seven sections (Sections 4–10) for preliminary assessment of the current Biorisk Management System at the RLEDP. The three sections not included in the checklist but are part of the standard are sections (1) scope, (2) normative references, and (3) terms and definitions.^17^ The MSBC consultant then prepared a schedule of activities to be completed in order to implement a Biorisk Management System in accordance with the ISO 35001:2019 requirements, which focused on the following sections:

- Section 4—Context of the Organization
- Section 5—Leadership
- Section 6—Planning
- Section 7—Support
- Section 8—Operation
- Section 9—Performance Evaluation
- Section 10—Improvement

As part of the gap analysis, the relevant information was obtained through the following methods:

- Discussions and interviews with managers and specialists from the RLEDP to clarify the specifics of managing the organization’s processes.
- Study of regulatory, technical, and legislative documentation that defines the processes and procedures for implementing activities and conducting applied research in the field of dangerous pathogens of animals including zoonoses, thus ensuring biosafety and biosecurity in the RLEDP.
- Interviews with specialists to analyze functional tasks and determine their role in increasing the efficiency of the current Biorisk Management System.
- Study of organizational and administrative documentation by defining the relationships, powers, responsibilities, and duties of all RLEDP employees.
- Study of internal RLEDP regulatory documentation on biosafety and biosecurity issues.

### Subject of Study

The subject of study was the RLEDP, which acquired new building facilities in 2016 through their collaboration with the Defense Threat Reduction Agency (DTRA) Biological Threat Reduction Program (BTRP). The building was designed and built based on the *Biosafety in Microbiological and Biomedical Laboratories* (*BMBL*), fifth edition, and the World Health Organization (WHO) International Laboratory Design and Maintenance.^24^ RLEDP operates as a structural subdivision of the Republican Veterinary-Sanitary and Phytosanitary Center of Laboratory Services (RVSPCLS) under the Food Safety Inspection Body of the Republic of Armenia.

The main priorities of RLEDP are primarily diagnostic, providing testing for infectious diseases of animals and performing all confirmatory testing for the satellite veterinary laboratories throughout the 10 regions of Armenia. RLEDP also performs scientific research and leads country-wide monitoring studies for infectious and invasive diseases of terrestrial and aquatic animals in Armenia. RLEDP has three subdivisions (departments): (1) Department of Necropsy and Histological Investigations; (2) Department of Microbiology and Parasitology; and (3) Department of Serology and Molecular Biology.

The RLEDP building comprises two floors with an area of 1500 m^2^, with facilities corresponding to the BSL-2 safety standards outlined in the *BMBL*, fifth edition.^25^ The building includes a basement and attic space, and the ground floor includes auxiliary elements including storage, laundry, dressing room, freezer, temporary tank for disinfection of liquid waste, server room for computer electronic systems, electrical panel and uninterruptible power supply rooms, water tank, system ventilation, offices, kitchen, and a conference room. Laboratory components on the ground floor include the necropsy room, sample reception room, and sterile media preparation room.

The diagnostic laboratories are located on the second floor of the building. This level has additional biosecurity and biosafety precautions including security systems with video surveillance in the laboratories and secure hallways that require keycard access, limiting admittance to appropriately trained staff with additional fingerprint access for storage of pathogens of concern to Armenia. Several laboratories are equipped with a negative air pressure system based on BSL-3 safety standards. The building is also equipped with a fire alarm system and emergency exits.

The laboratories are equipped with modern and maintained equipment and relevant diagnostic materials for accurate detection of various infectious diseases relevant to Armenia. Laboratory specialists and staff are trained regularly by various international organizations to maintain internationally recognized standards and provide updates for new technological capabilities.

The RLEDP has two electronic computer systems: Electronic Integrated Disease Surveillance System (EIDSS®) and Pathogen Asset Control System (PACS). EIDSS is a universal electronic system designed for disease registration, data exchange, controlled movement of samples, and traceability of work throughout Armenia. PACS is for the accounting, management, and control of biological agents stored at RLEDP. For safety and security, the systems are equipped with individual logins (login, password) and the delineation of rights and responsibilities based on different staff, which allows for clear restrictions of access to different types of stored information. All laboratory work is maintained in accordance with the diagnostic methods approved by the World Organization for Animal Health.^16^

## Instruments and Data Analysis

ISO 35001:2019 section checklist (Biorisk Management for Laboratories and Other Related Organizations) was used under the license, which was provided by the National Body of Standardization and Metrology on May 11, 2021. The gap analysis was conducted based on Sections 4–10 of the ISO 35001:2019 standards.^17^

## Preparation for Implementation of ISO 35001:2019

**Figure 1.**
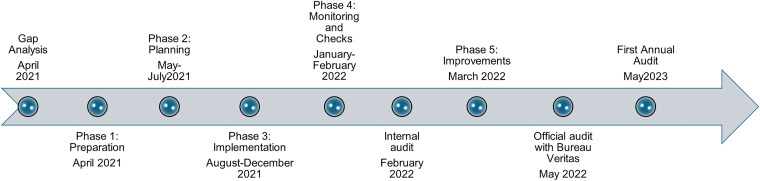
Timeline of Events to ISO 35001:2019 Certification.

Following the gap analysis, a multi-phased approach (five phases) was developed to address all necessary required elements for implementing a Biorisk Management Plan based on ISO 35001:2019. The elements to be addressed included the development of working groups, assignment of proper authorities and management and staff responsibilities, development of multiple documents for standardization of processes through virtual work and on-site trainings, and preparations over a 1-year period from April 2021 to March 2022 to prepare for the final audit by Bureau Veritas in May 2022. The year-long process was divided into five phases, including: (1) preparation, (2) planning, (3) implementation, (4) monitoring and checks, and (5) improvements (Figure 1).

Phase 4 included training RLEDP staff to perform an internal audit and Phase 5 included performance of all the corrective actions necessary following the internal audit. Additional on-site training was provided by a biosafety and biosecurity subject matter expert (SME) under the DTRA contract with Armenia, to fill the necessary gaps through 40 workshops and hands-on training events for biorisk management (Supplementary Table S1). These trainings are based on *BMBL* best practices and Global Biorisk Management Curriculum maintained by Sandia National Laboratories (https://gcbs.sandia.gov/tools/gbrmc/).

## Certification Audit by International Accreditation Agency Bureau Veritas

The certification process was carried out in two phases. Both phases were carried out remotely via teleconference using Microsoft Teams and occurred over 6–7 h each day of 3 days, 1 day for Phase 1 and 2 days for Phase 2. Phase 1 of the certification audit aimed to (1) check the documentation of the Client’s biorisk management system, (2) assess the location and site conditions and conduct discussions with personnel to determine their readiness for Phase 2, (3) study the client’s understanding of ISO35001:2019 standard, (4) gather information about the client’s processes and location, (5) review resource allocation for Phase 2, (6) confirm that the duration of the audit and planning time are taken into account and the client is prepared for this process, (7) planning of Stage 2 audit, and (8) assess client readiness based on documentation from their internal audit and review. Phase 1 also included verification of all appropriate documentation required for the standards and discussion of potential non-conformities. Phase 2, the final audit, occurred over a 2-day period. The objectives of the audit were (1) evaluate all management system standards and regulatory documents for compliance, (2) utilize key indicators to monitor, measure, record, and analyze performance of goals and objectives, (3) assess the client’s management system and activities with legal, regulatory, and contract requirements, (4) assess the process management of the client, (5) conduct internal audits and management reviews, and (6) evaluate management responsibility for the policies of the facility.

## Results

Based on the gap analysis, we have provided an overview of each section assessed with the accompanying recommendations and actions for compliance with ISO 35001:2019 standards and to successfully achieve the future accreditation. The results of the assessment scoring of the individual requirements within each section can be found in Table 1.

**Table 1.** Results of the implementation category scores of the gap analysis by section

ISO 35001:2019 standard sections	Implementation category score*^a^*
**Section 4—Context of the organization**	
4.1 Understanding the organization and its context	3
4.2 Understanding the needs and expectations of stakeholders	3
4.3 Determining the scope of the Biorisk Management System	2
**Section 5—Leadership**	
5.1 Leadership and commitment	2
5.2 Policy	3
5.3 Roles, responsibilities, and authorities in the organization	2
5.3.1 Top management	2
5.3.2 Senior management	2
5.3.3 Biorisk management committee	1
5.3.4 Biorisk manager advisor	2
5.3.5 Scientific management	2
**Section 6—Planning**	
6.1 Actions to address risks and opportunities	3
6.1.1 Hazard and/or threat identification and analysis	3
6.1.2 Risk assessment	3
6.1.3 Risk mitigation	2
6.1.4 Performance evaluation	3
6.2 Biorisk management objectives and plans to achieve them	3
**Section 7—Support**	
7.1 Resources	2
7.1.1 Employee health program	2
7.1.1.1 Vaccination of employees	3
7.2 Competence	2
7.2.1 Behavioral factors and employee management	2
7.2.2 Personnel reliability measures	2
7.3 Awareness	2
7.3.1 Training	2
7.4 Communication	2
7.5 Documented information	2
7.5.2 Creating and updating	2
7.5.3 Control of documented information	2
7.5.4 Information security	2
7.6 Non-employees	1
7.7 Personal safety and security	1
7.8 Supplier management	1
**Section 8—Operation**	
8.1 Operational planning and control	2
8.2 Commissioning and decommissioning	2
8.3 Maintenance, inspection, calibration, certification, and validation	2
8.4 Physical security	2
8.5 Biological materials inventory	2
8.6 Good microbiological technique	1
8.7 Clothing and personal protective equipment	1
8.8 Decontamination and waste management	2
8.9 Emergency response and contingency planning	2
8.9.1 Emergency scenarios	2
8.9.2 Emergency plan training	2
8.9.3 Emergency exercises and simulations	2
8.9.4 Contingency plans	2
8.10 Transport of biological materials	2
**Section 9—Performance evaluation**	
9.1 Monitoring, measurement, analysis, and evaluation	2
9.2 Internal audit	3
9.3 Management review	3
**Section 10—Improvement**	
10.1 General	2
10.2 Incident, non-conformity, and corrective action	2
10.3 Continual improvement	3

a Score 1 = activity/process is maintained in accordance with the requirements of the standard; score 2 = activity/process is maintained, but requires improvement; score 3 = activity/process is not performed in accordance with the requirements of the standard.

Implementation category scores were combined for each section from Table 1 by tallying the individual scores of standard sections to identify where the percentage of activities either complied or did not comply with the standard (Table 2). Based on the gap analysis scores on compliance of the RLEDP management system with the requirements of the ISO 35001:2019 standard, it was revealed that 89% (48/54) (Table 2) of the requirements of the standard had not been fully achieved, which required additional targeted activities to achieve compliance.

**Table 2.** Summary of the compiled implementation category scores (1, 2, 3) for RLEDP compliance based on the ISO 35001:2019 standard for each section

Standard section	Compiled assessment score		
	Activity in accordance with standard (1)	Partial concurrence/needs improvement (2)	Not in accordance with standard (3)
4 (4 subsections)	NA	1	3
5 (8 subsections)	1	6	1
6 (6 subsections)	NA	1	5
7 (16 subsections)	3	12	1
8 (14 subsections)	2	12	NA
9 (3 subsections)	NA	1	2
10 (3 subsections)	NA	2	1
Total	**6 (11%)**	**35 (65%)**	**13 (24%)**

NA or not applicable means there were no scores of 1 or 3 for that specific standard section.

RLEDP, Reference Laboratory for Especially Dangerous Pathogens.

### Section 4—Context of the Organization

RLEDP has a practice for identifying external and internal factors related to the strategic direction of the organization and can identify stakeholder’s requirements in relation to the quality management system. RLEDP activities involving biorisk management are regulated by international regulations (i.e., *BMBL*), as well as by the legislative requirements of the Republic of Armenia. There are current safety regulations, but the full list, including requirements and expectations, is incomplete. The processes required for inclusion in a Biorisk Management System are documented in existing standard operating procedures (SOPs) to some extent, but not all conform to the requirements of ISO 35001:2019.

#### Recommended Actions

- Develop documentation of the complete descriptions of all external and internal factors relevant to intentions and strategic direction for biorisk management.
- Develop documentation detailing the requirements and expectations of stakeholders in relation to biorisk management.
- Develop the scope and boundaries of a Biorisk Management System.
- Develop risk assessments for operating procedures.

### Section 5—Leadership

RVSPCLS management and the head of RLEDP demonstrated an understanding of the need to implement requirements based on international standards for biorisk management. Management has ensured biosafety and biosecurity by providing modern equipment and ensuring their use by qualified personnel using procedures developed by a biosafety committee. The developments of a Biosafety and Biosecurity Plan and a Biosafety Manual were also in development. While RVSPCLS manages the top management of RLEDP, the head of RLEDP oversees operational management of the laboratory and bears the primary responsibility for ensuring the biological safety of RLEDP.

#### Recommended Actions

- Define the responsibilities of senior management regarding the Biorisk Management System in the Biosafety and Biosecurity Guidelines.
- Establish a biorisk management committee and assign responsibilities based on the ISO 35001:2019 standard. The biorisk management committee should be defined in the Biosafety and Biosecurity Manual with rules to ensure the independence of committee members.
- Define the role of the Biorisk Management Consultant with assigned responsibilities based on the ISO 35001:2019 standard. These responsibilities should be defined in the Biosafety and Biosecurity Manual.
- Define scientific management responsibilities and functions in the Biosafety and Biosecurity Manual.

### Section 6—Planning

There were general biosecurity controls that were documented in SOPs based on international standards and best practices, but they were lacking in documented requirements, stating that controls should be included in design documents with the requirement to verify the effectiveness of existing controls. Additionally, identification and analysis of biorisks in the laboratory had not been provided, and no assessments of biorisks had been conducted. The biosafety controls that were documented in the SOPs were based on international standards and global best practices with controls related to biosecurity based on national requirements, legislation, international standards, and best global practices.

#### Recommended Actions

- Create, improve, and implement a unified, detailed methodology for assessing biorisks based on ISO 35001:2019 standards.

### Section 7—Support

RLEDP has created and operates a basic structure for ensuring biosafety and biosecurity through building and safety systems with defined SOPs covering biosafety in the laboratory. The requirements for understanding the working conditions of health professionals and medical examination of such personnel are provided in the following governmental decrees: (1) on approval of lists of professions with working conditions harmful to health, especially harmful and difficult conditions labor,^26^ and (2) on the procedure for conducting mandatory preliminary medical examination health status, the field of activity, those employed in are subject to a mandatory medical examination of their health status, a list of the scope and frequency of medical examinations and approval of forms of a personal sanitary (medical) record and a nominal list of persons subject to medical examination.^27^

Competency requirements are defined in employee job descriptions with competence ensured as part of the personnel management process. Staff development is maintained as part of the annually planned biosafety and biosecurity training.

General requirements for ensuring the reliability of personnel maintaining the confidentiality of information are established in the Republic of Armenia of Law on State and Official Secrets.^28^ RLEDP conducts training to ensure personal protection for employees, taking into account risks in the workplace. SOPs were previously developed under the quality management system in the RVSPCLS for this protection. Additional plans are being developed for staff training, both for new employees and to improve the competence of existing staff.

#### Recommended Actions

- Expand the laboratory biosafety and biosecurity framework to fully comply with ISO 35001:2019.
- Maintain records of medical surveillance on the health of laboratory personnel based on identified hazards and/or threats to the employee.
- Establish a vaccination program for laboratory staff.
- Add biological safety assessment parameters to each job description.
- Develop a detailed procedure for managing documented information, which meets all the requirements of the ISO 35001:2019 standard, and/or revise and supplement existing SOPs.
- Develop and implement an information security management system.

### Section 8—Operation

Operational planning in the organization is conducted in accordance with the planning process based on the internal requirements of the RVSPCLS. RLEDP department heads, together with the head of the RLEDP, plan and describe various work processes in facility SOPs, of which 15 were provided for the gap analysis. The planning process identifies and incorporates relevant legal requirements and incorporates information from industry’s best practices, but the requirements of risk assessments prior to starting work processes were lacking.

The commissioning of the RLEDP facility (validation) was carried out based on a risk assessment that complies with the requirements of national legislation, WHO International Laboratory Design and Maintenance,^24^ and best practices, but repeated revalidation of the building and systems has not been performed. A program for testing laboratory equipment in the RLEDP has been developed, but the framework for calibration and certification of equipment performed by accredited laboratories was not available. The building and property have appropriate working security controls with physical security provided by police officers with video surveillance, alarms, and warning systems.

The organization ensures that work is performed in accordance with proper microbiological techniques and appropriate personal protective equipment is provided to personnel. Waste disposal is performed by RVSPCLS with specific SOPs defined for RLEDP, but policy documentation was lacking. In addition, the RLEDP has developed an emergency action plan and a personnel evacuation plan. Regular staff training is conducted, and emergency exercises are also organized in the presence of representatives of the Ministry of Emergency Situations once or twice a year.

#### Recommended Actions

- Develop SOPs for all specific and general work processes, ensuring biological safety in the laboratory; specifically, develop the following documents:
  Biosafety and Biosecurity Manual
  o Biosafety and Biosecurity Plan
  o Regulation of the Committee on Biosafety and Biosecurity
- Revalidate the laboratory building systems in accordance with the requirements of the international laboratory construction and design standard, 28 in collaboration with a specialized foreign company.
- Provide calibration/certification of equipment in accordance with manufacturer and legal requirements as soon as the national regulations in Armenia are established.
- Ensure the rights of access to information from video surveillance, alarm, and warning systems to the head of the RLEDP or another authorized individual.
- Document a formal risk assessment as the basis for defining a process for reviewing, analyzing, updating, and reporting the presence of biological materials.
- Document the Waste Management Policy.
- Describe the measures for providing the continuity of work in unforeseen circumstances in the Biosafety and Biosecurity Plan.
- Develop the rules of transportation based on the results of a biorisk assessment.

### Section 9—Performance Evaluation

RLEDP conducts activities to monitor, analyze, and measure the state of biosafety and biosecurity. The method of monitoring measurement is indicated in specific SOPs for work processes based on a specific SOP. RLEDP also maintains SOPs for “Internal Audit of the Quality System” and “Management Review” under their quality system, but these SOPs are not based on biorisk management.

#### Recommended Actions

- Establish a formal process for monitoring, measuring, analyzing, and evaluating the Biorisk Management System, as well as establishing a documented procedure for reporting, identifying, documenting, analyzing, and investigating biorisk incidents.
- Develop and implement a specialized internal audit procedure for the Biorisk Management System or revise an existing SOP so that it fully complies with the requirements of the ISO 35001:2019 standard.
- Develop and implement a dedicated management review procedure for the Biorisk Management System or redesign an existing SOP so that it fully complies with the requirements of the ISO 35001:2019 standard.

### Section 10—Improvement

The management and employees of the RLEDP are focused on continuous improvement of their activities in the field of biosafety and ongoing improvements, including already existing procedures and the development of new SOPs. Within the framework of the RVSPCLS, the following three SOPs were previously developed: (1) control of non-conforming work, (2) corrective actions, and (3) preventive actions. RLEDP also provides timely responses to incidents and non-conformities by analyzing the causes and developing corrective actions.

#### Recommended Actions

- Develop specialized non-conformity and incident management and corrective action management procedures to be implemented within the Biorisk Management System, or update an existing SOP to comply with ISO 35001:2019.
- Reassess a biorisk assessment in the event of an incident or non-compliance.
- Develop SOPs for continual improvement.

 Based on the gap analysis results, a 1-year implementation period was set to create stable resources to satisfy the requirements and ensure the continuity of work. A total of 40 training courses were identified and delivered on topics such as development of a Biorisk Management System, incident response, and risk assessment (see Supplementary Table S1). Final documentation included the development of the following 11 site-specific documents:

(1) Biosecurity Plan
(2) Biosafety Plan
(3) Medical Waste Management Policy
(4) Sensitive Information Procedure
(5) Sensitive Information Policy
(6) Information Security Policy
(7) Biorisk Assessment Methodology
(8) Internal Audit of the Biorisk Management System
(9) Management of Non-Conformity
(10) Management of Corrective Action
(11) Analysis of the Biorisk Management System by Management

## Certification Audit

Stage 1 of the Certification Audit occurred on April 25, 2022. There were a few non-conformities that were noted in the Biorisk Management System documents that needed to be addressed prior to Phase 2 of the audit. Additionally, the organization had completed a full cycle of internal audits as required, the input data for the management review were provided in full as required, and the audit manager recommended to proceed to Phase 2 with the requirement that all non-conformities be addressed. The organization’s representative who participated in the audit was noted as demonstrating high competence, understanding, and motivation to achieve the positive results of Phase 1. Based on the Phase 2 audit, which occurred over a 2-day period, identified that the organization fully demonstrated the efficiency of the BRM and the management’s commitment to continuous improvement. The adequacy of corrective actions for the non-compliance was checked and confirmed. The RLEDP’s biorisk management system did meet the ISO 35001:2019 requirements.

## Discussion

In diagnostic laboratories working with biological hazards, there is always a risk of exposure to various unknown pathogens.^2,18,19^ Therefore, having an effective Biorisk Management System is essential to reduce and neutralize these risks. Working in an environment with unknown pathogens of different BSLs and potential zoonoses requires additional considerations, including restriction of certain procedures, such as pathogen isolation, to protect all levels of staff in the organization as well as the community where the laboratories reside. There are many forms of guidance to analyze and address biorisk management,^3,29–31^ and safe laboratory practices are outlined in the *BMBL* sixth edition,^4^ Laboratory Biosafety Manual fourth edition,^32^ and Biosafety and Biosecurity Manual^16^ to protect staff throughout an organization. The focus of this study was to bring laboratory and biorisk management practices up to international standards through the requirements and guidelines outlined in the ISO 35001:2019 standard following the PDCA principle.^17^ While this standard has been defined since 2019, in more recent years, laboratories are starting to re-evaluate their facilities by this standard, but even fewer are working to achieve this standard.^14,15^ Implementation of a standardized system and its full implementation is an important step to ensure effective biorisk management at an internationally recognized level of management and safety. This system includes providing a roadmap for others who are considering understanding and following this standard. In utilizing this standard, several gaps were identified that needed to be addressed and have highlighted the targeted work required to achieve certification. We also identified some challenges at the beginning of this process. RVSPCLS management was initially hesitant to move forward with the implementation process due to the ongoing cost of certain specific laboratory consumables and the periodic audits but once they understood the importance of maintaining a BRM and its safety for all staff and the public, it became clear that this was an important investment. Some staff were also hesitant in that they were concerned it would cause an undue burden on their workload. Once they understood that they were already doing most of the work, that their changes would involve doing things in a more systemized way with documentation, they realized it was not that difficult to implement.

We also want to emphasize that to reach this certification, we were fortunate to have international training assistance and funding for this process. Specifically, our prior formalized biosafety and biosecurity trainings and accumulated practical experience at RLEDP, combined with improved personnel qualifications and available documentation that was ready to be refined and systemized, served as the basis for the successful development and implementation of a Biorisk Management System that meets the requirements of the ISO 35001:2019 standard. Without some of the building and laboratory equipment improvements, prior trainings, and updates that have occurred at the facility, it would have required more time to accomplish this goal of standardization. There are also available resources, guidelines, and companies that can assist with this process. We have highlighted the two that we worked with that could lend their expertise to others. Laboratories need to understand that there will be upfront costs to get a laboratory into the proper space for this certification, and there will be ongoing costs to be considered with future upkeep and audits. It’s important to get management and all staff on board prior to this undertaking so that it can be maintained over time. Even with these improvements, the gap analysis defined by the ISO 35001:2019 standard checklist identified gaps in seven assisted sections of the standard, which required the organization and delivery of 40 training courses. These additional courses were delivered through a DTRA BTRP program in Armenia that utilized an international SME in biosafety and biosecurity to provide training to the appropriate staff to meet the requirements of the standard. This process also included guidance from MSBC consultants who performed the gap analysis, prepared the schedule of activities, and guided RLEDP through the process with final evaluation and feedback prior to the official audit.

Gaps were identified that the organization still needs to improve upon as part of the implementation of the Biorisk Management System. RLEDP needs to review the necessary processes that form the basis of the Biorisk Management System and maintain documentation on the sequence of these processes. In addition, the organization needs to create and implement a unified biorisk assessment methodology across all staff with specific responsibilities attributed to specific members of staff and senior management during the implementation processes. This process requires that the roles and responsibilities of the Biorisk Management Consultant and the Biorisk Management Committee be clearly defined in the Biosafety and Biosecurity Manual for RLEDP. Human resources is also a valuable part of the team as a cornerstone of any organization. Their participation is necessary to oversee and ensure that the continuous training of personnel is regularly maintained so that RLEDP can successfully perform the necessary activities as defined by the requirements of the ISO 35001:2019. In addition, the organization needs to implement an appropriate occupational health plan to cover medical surveillance and vaccinations of laboratory personnel and staff, where appropriate. Overall, we feel this certification has real-world implications for Armenia and other countries that may be considering this certification. This was an opportunity to improve the safety of our laboratory, our staff by emphasizing the focus on risks and maintaining an environment that reduces those risk to ourselves and the population as a whole. We feel that this will have an impact on the quality of our diagnostic work through the documented processes that maintain our consistency and the reduction of overall risks that can be maintained through these processes.

While the initial development and implementation of the Biorisk Management System is the foundation of a successful program, the processes that are in place need to be maintained and updated as necessary to retain accreditation. RLEDP must continue to maintain the requirements and strive for continuous improvement of the suitability, adequacy, and effectiveness of the Biorisk Management System. It is necessary to enhance the biorisk management performance by implementing a culture of ongoing, long-term processes to support the Biorisk Management System by promoting the participation of all staff in both implementation and execution. It will be important to share and discuss the relevant results on execution with both staff and other relevant stakeholders to successfully monitor, record, and maintain the relevant documentation as evidence of ongoing improvement. This process requires the formation of teams including personnel involved in improving business processes and regularly training team members on methods for continuous improvement. Senior management is essential to the process of implementing and maintaining a Biorisk Management System. Senior management must understand the importance and advantages of compliance, realize the consequences of operating without a Biorisk Management System, and provide the financial resources to maintain the system through facilities management and appropriately trained staff. Interdepartmental collaboration, specifically with the Ministry of Health, is also important and needs to be improved, as during the implementation of any new system, legislative gaps can often be discovered that may affect compliance with the requirements of the standard. Specifically, the requirements of Section 7 Clause 7.1 (Employee Health Program and Vaccination of Employees) were not met by the organization based on legislative regulations that are not yet in place to provide this type of program for RLEDP.

Identified gaps related to the employee health program are quite common. Similar problems were identified during the gap analysis in the implementation of the Biorisk Management System in the laboratories of the clinical and medical referral centers in Indonesia.^14,15^ In addition, measures for staff reliability must also be considered, as significant gaps in this section could be detrimental for the laboratory.

Biosafety and biosecurity are important elements of public health systems in addressing global health challenges related to biological hazards.^2,5,6^ A specific law on biosafety and biosecurity is under development in Armenia, which will provide guidance and set standards based on international rules and requirements to regulate the management of biorisks and safety in Armenia, while complying with international standards.

Biosafety and biosecurity is an ongoing commitment that all laboratories need to maintain and prioritize to fully understand the potential dangers that can occur in laboratories and affect laboratory personnel. The consequences of accidental release outside of the laboratory^18,20,22^ should be fully understood by all parties involved, and adherence to a Biorisk Management System can provide the framework to ensure the safety of individuals and keep pathogens secure.

Following 1 year of targeted and focused work, RLEDP was able to implement a Biorisk Management System, and the laboratory achieved ISO 35001:2019 certification in 2022. While certification is an important step, the importance of long-term plans for maintenance and compliance must be considered to maintain the proper level of biorisk management in the future. The entire laboratory staff, including management, value this achievement and are constantly working to maintain and continuously improve the system. This vigilance and support resulted in the laboratory successfully passing the first annual audit in 2023 to retain certification of this standard, first achieved in 2022.

## Authors’ Contributions

H.D.: Conceptualization, data curation, methodology, writing—original draft, writing—review and editing. P.T.: Conceptualization, supervision, methodology, writing—review and editing.

## Supplemental Material

sj-docx-1-apb-10.1089_apb.2025.0007 — Supplemental material for Implementation of a Biorisk Management System in Armenia for ISO 35001:2019 Certification Supplemental material, sj-docx-1-apb-10.1089_apb.2025.0007 for Implementation of a Biorisk Management System in Armenia for ISO 35001:2019 Certification by
