# Supplementary material for: Implementation of a Biorisk Management System in Armenia for ISO 35001:2019 Certification
Source: Appl Biosaf. 2025 Apr 21;31(2):80–91. doi: 10.1089/apb.2025.0007 (PMC13201930; doi:10.1089/apb.2025.0007)
Supplement: sj-docx-1-apb-10.1089_apb.2025.0007 — Supplemental material for Implementation of a Biorisk Management System in Armenia for ISO 35001:2019 Certification [file sj-docx-1-apb-10.1089_apb.2025.0007.docx]

**Supplementary Table 1.** List of trainings conducted during the ISO 35001:2019 implementation period.

| **Training Topic** |
| --- |
| 1.Development and Implementation of a Biorisk Management System (based on the requirements of ISO 35001:2019) |
| 2.Biorisk Management (in accordance with ISO 35001:2019 standard) |
| 3.Biorisk Management System Internal Audit (according to the requirements of ISO 35001:2019 and ISO 19011:2018) |
| 4. Personnel Reliability Program Reporting Requirements and Malicious Insider Threats (in accordance with ISO 35001:2019 standard) |
| 5. Especially Dangerous Pathogens (EDPs) and Emergency Response (in accordance with ISO 35001:2019 standard) |
| 6. Identifying Legal Requirements that Impact Biorisk Management |
| 7. Establishing and Maintaining Worker Health Programs |
| 8. Incident Response Planning and Preparation |
| 9. Incident Response and Investigation |
| 10. Incident Response Evaluation and Improvement |
| 11. Good Laboratory Work Practices |
| 12. Personal Protective Equipment |
| 13. Disinfection and Decontamination |
| 14. Biological Waste Disposal |
| 15. Shipping Infectious Substances and Biological Specimens |
| 16. Laboratory Biosecurity |
| 17. Field Biosecurity |
| 18. Biocontainment Facility Features |
| 19. Engineering Controls and Laboratory Equipment |
| 20. Managing Human Performance in the Biorisk Management Workforce |
| 21. Hazard and Risk Communication in the Laboratory |
| 22. Understanding and Maintaining Facilities and Equipment for Biorisk Management |
| 23. Basic Features and Maintenance for Physical and Information Security Measures |
| 24. Conducting Audits and Inspections to Assess Biorisk Management Performance |
| 25. Biosafety Risk Assessment |
| 26. Biorisk Mitigation Strategies |
| 27. Biorisk Characterization and Evaluation |
| 28. Administrative Control for Biorisk Management |
| 29. Writing and Communicating Biorisk Management Policy |
| 30. Introduction to Dual-Use Research of Concern |
| 31. Developing and Maintaining an Inventory Management System |
| 32. Managing Access To, Control Of, and Accountability for Biological Materials |
| 33. Incident Management: Pre-Event Activities |
| 34. Introduction to Emergency Response Planning |
| 35. Biological Spill Clean up |
| 36. Emergency Response Planning – Tabletop Exercise/Scenario Development |
| 37. Live Emergency Response Drill – Spills in the Laboratory |
| 38. Live Emergency Response Drill – Medical Emergencies |
| 39. Live Emergency Response Drill – Fire Drill |
| 40. Live Emergency Response Drill – Outside Emergency Responders |
